# Antinociceptive effects of lacosamide on spinal neuronal and behavioural measures of pain in a rat model of osteoarthritis

**DOI:** 10.1186/s13075-014-0509-x

**Published:** 2014-12-23

**Authors:** Wahida Rahman, Anthony H Dickenson

**Affiliations:** Department of Neuroscience, Physiology and Pharmacology, University College London, Gower Street, London, WC1E 6BT UK

## Abstract

**Introduction:**

Alterations in voltage-gated sodium channel (VGSC) function have been linked to chronic pain and are good targets for analgesics. Lacosamide (LCM) is a novel anticonvulsant that enhances the slow inactivation state of VGSCs. This conformational state can be induced by repeated neuronal firing and/or under conditions of sustained membrane depolarisation, as is expected for hyperexcitable neurones in pathological conditions such as epilepsy and neuropathy, and probably osteoarthritis (OA). In this study, therefore, we examined the antinociceptive effect of LCM on spinal neuronal and behavioural measures of pain, *in vivo*, in a rat OA model.

**Methods:**

OA was induced in Sprague Dawley rats by intraarticular injection of 2 mg of monosodium iodoacetate (MIA). Sham rats received saline injections. Behavioural responses to mechanical and cooling stimulation of the ipsilateral hind paw and hindlimb weight-bearing were recorded. *In vivo* electrophysiology experiments were performed in anaesthetised MIA or sham rats, and we recorded the effects of spinal or systemic administration of LCM on the evoked responses of dorsal horn neurones to electrical, mechanical (brush, von Frey, 2 to 60 *g*) and heat (40°C to 50°C) stimulation of the peripheral receptive field. The effect of systemic LCM on nociceptive behaviours was assessed.

**Results:**

Behavioural hypersensitivity ipsilateral to knee injury was seen as a reduced paw withdrawal threshold to mechanical stimulation, an increase in paw withdrawal frequency to cooling stimulation and hind limb weight-bearing asymmetry in MIA-treated rats only. Spinal and systemic administration of LCM produced significant reductions of the electrical Aβ- and C-fibre evoked neuronal responses and the mechanical and thermal evoked neuronal responses in the MIA group only. Systemic administration of LCM significantly reversed the behavioural hypersensitive responses to mechanical and cooling stimulation of the ipsilateral hind paw, but hind limb weight-bearing asymmetry was not corrected.

**Conclusions:**

Our *in vivo* electrophysiological results show that the inhibitory effects of LCM were MIA-dependent. This suggests that, if used in OA patients, LCM may allow physiological transmission but suppress secondary hyperalgesia and allodynia. The inhibitory effect on spinal neuronal firing aligned with analgesic efficacy on nociceptive behaviours and suggests that LCM may still prove worthwhile for OA pain treatment and merits further clinical investigation.

## Introduction

Osteoarthritis (OA) is a degenerative joint disease that is most prevalent in the elderly, and it is the primary source of disability and one of the largest health care burdens in the Western world [1]. Given the trend towards an increasing elderly and obese (excessive weight is a risk factor) population, the socioeconomic cost of OA is likely to rise.

OA is characterized by damage to the articular cartilage and subchondral bone, as well as episodic inflammation of the joint. Its clinical presentation is dominated by pain in the area surrounding the joint and often in areas distant to the affected joint (referred pain/secondary hyperalgesia) [2-6]. Current analgesic regimens include paracetamol, nonsteroidal anti-inflammatory drugs, opioids, steroids and combinations thereof, but they are unsatisfactory for many patients, due either to inadequate pain relief and/or to intolerable side effects [7]; thus, a large unmet clinical need remains. Therefore, unravelling the pathological mechanisms underlying the pain state is of major clinical importance in the development of more clinically effective drugs.

The transmission of pain from the peripheral site of injury, beyond the peripheral transducers, requires activation of voltage-gated sodium channels (VGSCs) located on peripheral nociceptors, and abundant data exist showing that alterations in the functional activity, distribution and density of VGSCs are critical for mediating chronic pain in both animals and humans [8,9]. Current health care guidelines do not recommend sodium channel blockers for the treatment of OA pain; however, there is strong evidence for abnormal firing in peripheral and central neurones in the arthritic condition, which must involve alterations in VGSCs [10-17], and a genetic mutation in the encoding gene for the 1.7 sodium channel subtype has been correlated with increased pain sensitivity in OA patients [18]. Indeed, the lidocaine patch and intraarticular injection of nonselective VGSC blockers has been shown to provide pain relief in patients with OA [19-21]; therefore, there is a rational basis for extending their application to the OA pain state.

One strategy that is being explored is widening the application of existing licensed anticonvulsants for the treatment of chronic pain [22,23]. The reasons for this are twofold. First, epilepsy and chronic pain share common neuronal mechanisms of increased excitability, providing a logical mechanistic basis, and anticonvulsant drugs with sodium channel–blocking activity, such as carbamazepine and phenytoin, have efficacy in chronic pain associated with nerve injury [24], and it is thought that OA pain may include a neuropathic component [25-28]. Second, preclinical and patient data already exists with regards to toxicity and tolerability of the drug in the setting of epilepsy, which could be built upon to improve clinical trials. One such anticonvulsant of interest is lacosamide (LCM), which is licensed for the treatment of partial onset seizures. Despite a recent negative outcome in phase III trials for the treatment postherpetic neuralgia (PHN) [22], the potential for LCM in treating OA pain, and indeed other types of chronic pain (including nerve injury pains other than PHN), may still exist because there is an argument that different sets of mechanisms may cause pain in neuropathic pain patients and also anecdotal evidence of neuropathic patients who respond to one analgesic over another [29].

LCM is a novel drug that targets the slow, inactivated state of VGSCs to promote time spent in the refractory state and hence directly reduce neuronal firing rate. The slow inactivation state of neurones is induced under conditions of repetitive neuronal firing and/or under conditions of slight or sustained membrane depolarisation. This is likely to be the case for the OA condition because spontaneous and enhanced evoked activity of joint nociceptors and dorsal horn neurones have been reported [10-17], as has sensitisation of spinal nociceptive withdrawal reflexes [30], indicating that the monosodium iodoacetate (MIA) model is associated with hyperexcitability. Given the aforementioned evidence for altered primary afferent activity in this model and the fact that blocking sensory fibre inputs with local anaesthetics blocks OA pain [21], our aim was to study the effects of LCM on spinal neuronal activity evoked from stimuli applied to the ipsilateral hind paws of rats using *in vivo* electrophysiological techniques. These techniques allow spinal nociceptive processing and central sensitisation to be studied experimentally and provide information on suprathreshold responses, which are likely to equate to high levels of pain transmission as reported by patients, as compared with behavioural data on the basis of which the analgesic effect of drugs on threshold responses are generally measured. The effects of systemic administration of LCM on behavioural measures of hypersensitivity were also tested.

## Methods

Sprague Dawley rats (Central Biological Services, University College London, UK), weighing 130 to 150 g at the time of model induction and 250 to 270 g at the time of *in vivo* electrophysiology and behavioural pharmacology, were employed for this study. All experimental procedures were approved by the UK Home Office and were carried out in accordance with the guidelines of the International Association for the Study of Pain [31].

### Induction of osteoarthritis

On day 0, isoflurane-anaesthetised Sprague Dawley rats weighing 130 to 150 g received an intra-articular injection of 2 mg of MIA in 25 μl of 0.9% saline through the infrapatellar ligament of the knee. Sham animals received injections of sterile 0.9% saline only. Following the injections, the animals were allowed to recover and then were rehoused in cages under a 12-hour alternating light–dark cycle with *ad libitum* access to food and water. Animal welfare was monitored over the 2-week period after model induction, with mobility and weight gain seen to be normal.

### Assessment of pain-related behaviour

Animals were habituated to the test environment for at least 30 minutes prior to model induction and behavioural testing. Behavioural assessment of hypersensitivity was carried out on day 7 and day 14 postinjection. The assessor was blinded to the model and treatment for the animals used in the behavioural pharmacology study, but not for the animals used in the *in vivo* electrophysiology study; however, here, quantification and recording of neurones provide an entirely objective measure, as does the effect of the drug on these measures.

#### Hind limb weight-bearing

Changes in hind paw weight-bearing were measured using an incapacitance tester (Linton Instrumentation, Norfolk, UK). Animals were placed in a Perspex (poly(methyl methacrylate)) chamber designed to keep the animal upstanding while the hind paws rest on a separate small electronic balance so that the weight distributed on the right and left hind paws can be measured. Once the animal was settled, three consecutive readings (each measured over 3 seconds) were recorded. The average of a total of three readings was determined for each hind limb for each rat and used for subsequent analyses. The weight-bearing of the ipsilateral hind paw to knee injection is presented as a percentage of the total weight-bearing of both hind limbs.

#### Development of mechanical and cooling hypersensitivity

Behavioural responses to stimulation of the ipsilateral hind paw were recorded once the animals had acclimatized to the testing area (Perspex cages with a wire mesh floor) for at least 30 minutes. Tactile hypersensitivity was tested by touching the plantar surface of the hind paw with von Frey (vF) filaments (Touch-Test; North Coast Medical, Gilroy, CA, USA) using the ‘up–down method’ [32], starting with 2.0 *g* then ranging from 0.4 *g* to 15 *g*. A cutoff of 15 *g* was set. Positive withdrawals were counted as biting, licking and withdrawal during or immediately following the stimulus. The strength of the vF filament was increased or decreased following a negative or positive response, respectively. This up–down procedure was applied four times following the first change in response. Data are presented as 50% paw withdrawal threshold (PWT) for each group ± standard error of the mean (SEM). Sensitivity to cooling stimulation was assessed as the number of withdrawals out of a trial of five applications of a drop of acetone to the plantar surface of the ipsilateral hind paw. Withdrawal frequency was quantified and presented as a percentage of the maximal response calculated as (number of foot withdrawals/5 trials) × 100.

### Pharmacological studies

#### *In vivo* electrophysiology

Twenty-four animals were used in this study, with 12 allocated per group (sham or MIA injection). Two weeks after MIA or sham injection, *in vivo* electrophysiological studies were performed (post-MIA injection days 15 and 16) as previously described [33]. Briefly, animals were initially anaesthetised with isoflurane (4%) delivered in a gaseous mix of N_2_O (66%) and O_2_ (33%). A laminectomy was then performed with the animals under isoflurane anaesthesia of 2% to 3% to expose the L4-L5 segment of the spinal cord. Anaesthetic levels of 1.5% to 1.7% were maintained for the duration of the experiment. Extracellular recordings were taken from ipsilateral deep dorsal horn neurones (lamina V-VI) using parylene-coated Tungsten electrodes (A-M Systems, Carlsborg, WA, USA). All the neurones recorded in this study were wide dynamic range neurones, because they all responded to both light touch and noxious inputs (pinch and noxious heat). Furthermore, all neurones responded to natural stimuli in a graded manner with coding of increasing intensity.

The evoked response to a train of 16 transcutaneous electrical stimuli (2-millisecond-wide pulses, 0.5 Hz) applied at three times the threshold current for C-fibre activation of the dorsal horn cell. The train of electrical stimuli was delivered via stimulating needles inserted into the peripheral receptive field, following which a poststimulus histogram was constructed. Responses evoked by Aβ- (0 to 20 milliseconds), Aδ- (20 to 90 milliseconds) and C-fibres (90 to 350 milliseconds) were separated and quantified on the basis of latency. Responses occurring after the C-fibre latency band were taken to be the postdischarge of the cells (350 to 800 milliseconds). Two other measures of electrically evoked neuronal activity were made. One was the ‘input’, which is calculated as the number of action potentials evoked by the first stimulus (due to incoming C-fibre activity) in the train of electrical stimuli response multiplied by 16. Thus, ‘input’ is a measure of the nonpotentiated response—that is, the baseline C-fibre-evoked response, which is likely a measure of afferent input and the resultant spinal neuronal response prior to central neuronal hyperexcitability evoked by subsequent stimuli. We also measured ‘wind-up’, which is calculated as the total number of action potentials evoked by C-fibre activity, minus the input. This potentiated response, seen as increased neuronal activity in response to constant repetitive C-fibre stimulation, is a model of temporal summation and central sensitisation. The centre of the peripheral receptive field was also stimulated using mechanical punctate and thermal stimuli (vF filaments, 2, 8, 26 and 60 *g*; and heat, applied with a constant water jet, 40°C, 45°C and 48°C). Application of each vF hair was separated by a minimum interval period of 5 to 10 seconds, and longer for very responsive neurones at the higher intensity range. Application of each subsequent heat stimulus was separated by a minimum period of 1 minute. All natural stimuli were applied for a period of 10 seconds per stimulus. Data were captured and analysed by a CED Power1401 interface coupled to a Pentium microprocessor with Spike2 software (PSTH and rate functions; Cambridge Electronic Design, Cambridge, UK). The data for the vF forces are expressed on a linear scale because the aim was to investigate drug effects on different intensities of stimuli.

Pharmacological assessment was carried out on only one neurone per animal. The testing procedure was carried out every 20 minutes and consisted of a train of electrical stimuli followed by natural stimuli as described above. Following three consecutive stable control trials (<10% variation for the C-fibre-evoked response and <20% variation for all other parameters), neuronal responses were averaged to give the predrug control values. LCM was then administered either via topical spinal application (10, 50 and 100 μg/50 μl) or systemically via subcutaneous injection into the scruff of the neck (3 and 30 mg/kg). The effect of each dose was followed for 1 hour, with tests carried out at 10, 30 and 50 minutes before the next dose was applied cumulatively.

#### Behaviour

Twenty animals were employed in this study; five animals received an intra-articular injection of saline and fifteen received MIA. Development of behaviour was assessed on days 7 and 14 postinjection, when animals were randomly allocated to a Perspex chamber, and, following a 30-minute acclimatization period in the testing room, baseline predrug behaviour was recorded as described above, with the assessor blinded to the model. Only animals that received MIA injections developed significant behavioural hypersensitivity and were used to study the effects of vehicle or LCM injection. Animals received an intraperitoneal injection of saline (vehicle) or 3 or 30 mg/kg LCM (*n* = 5 per group) and randomly allocated to a Perspex chamber. The experimenter was blinded to the injections. Behavioural tests were performed at 30 and 60 minutes postinjection, always following the same sequence for each batch of experiments: acetone tests for all rats in order would be interspersed in the interval between each PWT, that is, acetone test rats 1 − *x*, PWT rat 1, acetone test rats 1 − *x*, PWT rat 2 and so forth. Animals were then placed in the incapacitance tester, and hind limb weight-bearing was recorded as detailed above.

#### Statistical analysis

All statistical tests were performed on raw data using SPSS v22 software (IBM, Armonk, NY, USA). All data are presented as mean ± SEM. For *in vivo* electrophysiology measurements, statistical significance was tested using a nonparametric Mann-Whitney *U* test to compare two groups of data, and a one-way or two-way repeated-measures analysis of variance (RM-ANOVA), followed by Bonferroni-corrected paired *t*-tests, was performed when simultaneously comparing more than two groups of data. Drug effects were measured as the maximum percentage change from the averaged predrug control values for each dose on each measure per neurone. The overall effect of the drug (raw value) was then expressed and presented as the mean maximal evoked neuronal response for each dose. A one-way RM-ANOVA was used to evaluate drug effects on the neuronal responses evoked by electrical and dynamic brush stimulation, and a two-way RM-ANOVA was used to evaluate drug effects on the neuronal responses evoked by mechanical or heat stimulation in MIA or control rats. Behavioural data were analysed using the Mann-Whitney *U* test to compare two groups of data; to compare more than two groups of data, the Kruskal-Wallis test, followed by Dunn’s posttest, was used. Values of *P* < 0.05 were considered significant.

## Results

### Development of behavioural hypersensitivity after monosodium iodoacetate injection

**Figure 1:**
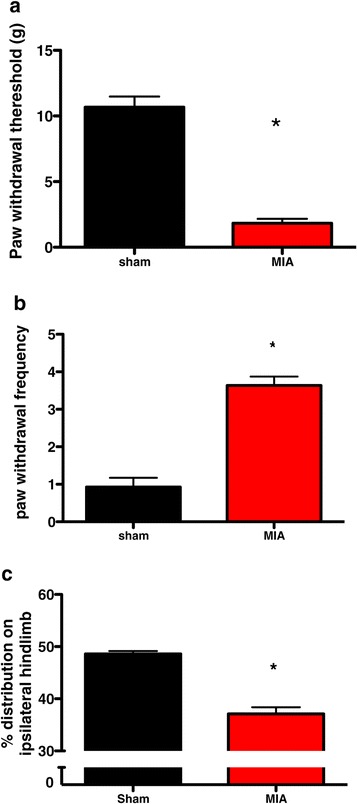
**Behavioural assessment at day 14 after monosodium iodoacetate (** ***n*** **= 27) or saline injection (** ***n*** **= 17).** Monosodium iodoacetate (MIA injection) into the knee resulted in behavioural hypersensitivity, compared with the sham controls that received saline injections, as evidenced by the significant reduction in the paw withdrawal threshold to mechanical punctate stimulation **(a)**, an increased paw withdrawal frequency to acetone application **(b)** and a decrease in the amount of weight borne on the hind limb ipsilateral to injection **(c)**. *P < 0.05 compared with shams by Mann-Whitney *U* test. Values are means ± SEM.

Hypersensitivity to mechanical and cooling stimuli (akin to allodynia) was observed in the MIA group. This was seen as a significant decrease in the ipsilateral PWT in the MIA group (Figure 1a). In addition, a significant increase in the frequency of paw withdrawals to cooling stimulation was seen in MIA rats compared with the same stimulation of the ipsilateral hind paw in sham controls, which elicited few, if any, hind limb withdrawals (Figure 1b).

A shift in hind paw weight-bearing was observed in the MIA group, with less weight distributed onto the right osteoarthritic limb compared with the left (contralateral) hind limb. In comparison, the distribution of weight in the sham control rats was largely equal over both hind limbs, indicating the presence of ongoing joint discomfort and/or pain in the MIA rats only (Figure 1c).

### *In vivo* electrophysiology-evoked responses of dorsal horn neurones

**Figure 2:**
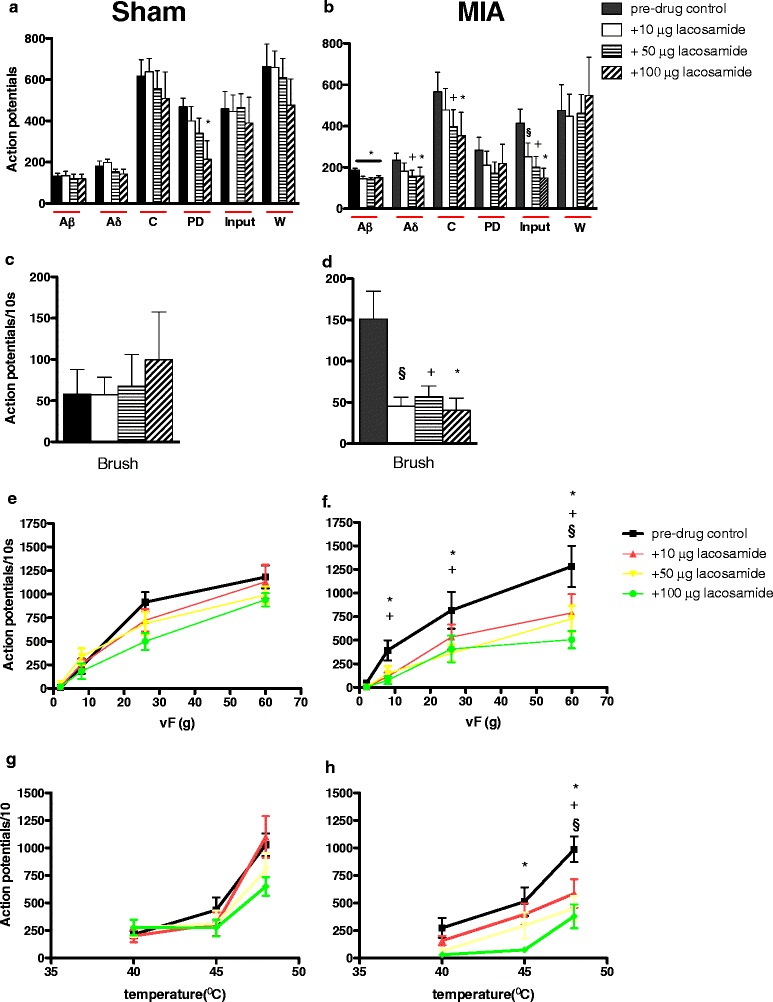
**Comparison of the effects of spinal administration of lacosamide and monosodium iodoacetate.** These graphs show the effects of lacosamide (LCM; 10, 50 and 100 μg) on the evoked neuronal responses to electrical **(a, b)**, dynamic brush **(c, d)**, mechanical punctate **(e, f)** and thermal stimulation **(g, h)** of the peripheral receptive field in sham rats (*n* = 6, left panel) and monosodium iodoacetate (MIA) rats (*n* = 7, right panel). The neuronal responses evoked by Aβ-, Aδ- and C-fibres and the input measure of neuronal excitability, dynamic brush, von Frey (vF) 8 to 60 *g* and 45°C to 48°C heat stimulation were significantly reduced by LCM in the MIA group. LCM did not produce any significant effect on any neuronal measure in the sham group. Asterisks and bars denote statistically significant main effects (one-way repeated-measures analysis of variance (RM-ANOVA)). § denotes significance at 10 μg, + denotes significance at 50 μg and * denotes significance at 100 μg compared with baseline control data (*P* < 0.05 by one-way RM-ANOVA with Bonferroni-corrected paired *t*-test). Values are mean ± SEM. PD, Postdischarge; Input, Baseline C-fibre-evoked response measure of afferent input and the resultant spinal neuronal response prior to central neuronal hyperexcitability evoked by subsequent stimuli; W, Wind-up, a frequency-dependent incremental increase in neuronal response to repetitive stimulation of C-fibres (that is, a measure of central neuronal hyperexcitability).

**Figure 3:**
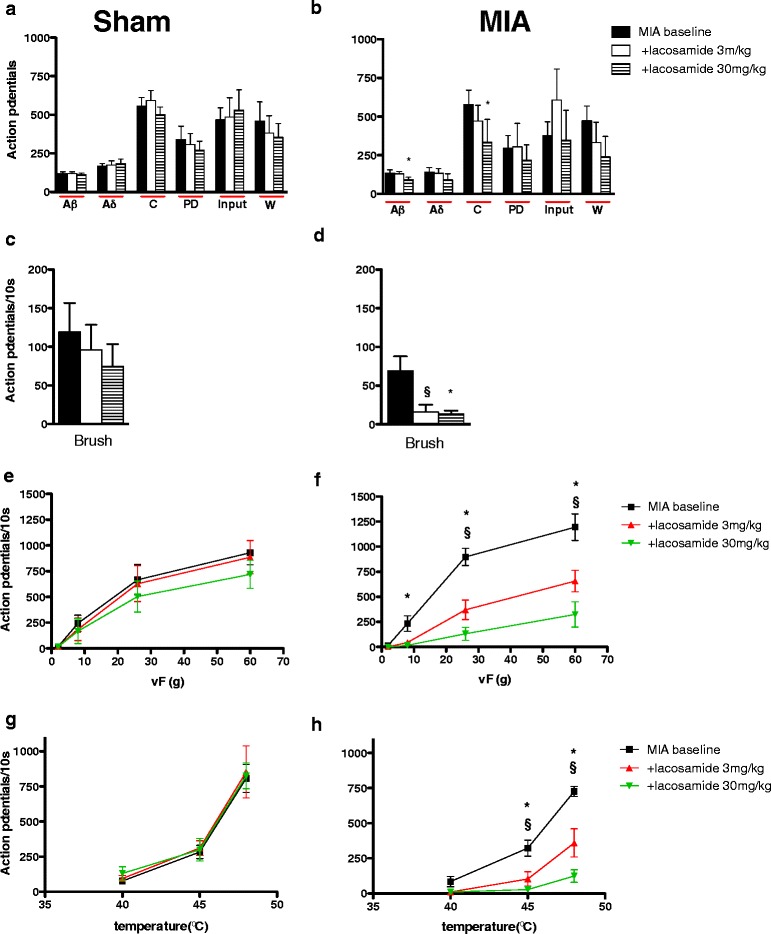
**Comparison of the effects of systemic administration of lacosamide and monosodium iodoacetate.** These graphs show the effects of lacosamide (LCM; 3 and 30 mg/kg) on the evoked neuronal responses to electrical **(a, b)**, dynamic brush **(c, d)**, mechanical punctate **(e, f)** and thermal stimulation **(g, h)** of the peripheral receptive field in sham rats (*n* = 6, left panel) and monosodium iodoacetate (MIA) rats (*n* = 5, right panel). Neuronal responses evoked by Aβ- and C-fibres, dynamic brush, von Frey (vF) 8 to 60 *g* and 45°C to 48°C heat stimulation were significantly reduced by LCM in the MIA group only. Asterisks and bars denote statistically significant main effect (one-way repeated-measures analysis of variance (RM-ANOVA)). § denotes significance at 3 mg/kg, and * denotes significance at 30 mg/kg, compared with baseline control data (*P* < 0.05 by one-way RM-ANOVA with Bonferroni-corrected paired *t*-test). Values are mean ± SEM. PD, Postdischarge; Input, Baseline C-fibre-evoked response measure of afferent input and the resultant spinal neuronal response prior to central neuronal hyperexcitability evoked by subsequent stimuli; W, Wind-up, a frequency-dependent incremental increase in neuronal response to repetitive stimulation of C-fibres (that is, a measure of central neuronal hyperexcitability).

The effect of LCM delivered via a spinal or systemic route was assessed upon the evoked responses of deep dorsal horn (lamina V-VI) neurones to electrical and natural stimulation of their peripheral receptive fields. Comparison of the average baseline predrug responses for MIA and shams per route of administration (spinal or systemic) revealed a significantly greater Aβ-fibre- and brush-evoked response in the MIA versus sham animals in the ‘spinal’ study (*P* < 0.05 by Mann-Whitney *U* test) (Figures 2a to 2d), but a significantly greater postdischarge in the sham group versus the MIA group (*P* < 0.05 by Mann-Whitney *U* test) (Figures 2a and 2b). However, no significant difference was seen with any of the electrical or brush-evoked responses in the ‘systemic’ study (*P* > 0.05 Mann-Whitney *U* test) (Figures 3a to 3d). No significant difference was seen between the vF- and heat-evoked response in MIA versus sham rats in either study (*P* > 0.05 by two-way ANOVA with Bonferroni posttest) (Figures 2e though 2h and Figures 3e through 3h). The relatively small sample size of neurones per group and per route of administration means that the mean can on occasion be shifted dramatically. Furthermore, as the present study was not powered to compare baseline neuronal responses between the MIA and sham groups, any differences in the average baseline neuronal responses were not further analysed or emphasized. It should be noted, however, that when we have previously characterized a large number of cells we observed, on average, greater firing of neurones in response to mechanical and thermal stimulation in the MIA group, but not to electrical or brush stimuli [15].

#### Effects of spinal administration of lacosamide

Topical spinal application of LCM produced a dose-related inhibition of a number of electrical and natural evoked neuronal responses. LCM inhibited the Aβ- Aδ- and C-fibre-evoked neuronal responses in the MIA group only. In addition, LCM produced a dose-dependent inhibition of the input measure of neuronal activity (Figure 2b), which is suggestive of actions on afferent activity as well as spinal transmission. Marked inhibitions of the natural evoked responses in the MIA group were seen, with all three doses producing an equivalent degree of significant inhibition of the neuronal response to brush stimulation (Figure 2d) and the neuronal response evoked by 60 *g* stimulation (Figure 2f). The evoked responses to 8 and 26 *g* were inhibited by only the top two doses of LCM (50 and 100 μg) (Figure 2f). LCM inhibited the evoked response to noxious heat stimulation at 45°C and 48°C, whereas the response evoked by innocuous heat stimulation (40°C) of the hind paw was not affected by LCM at any dose level (Figure 2h).

LCM produced minor inhibitions of some of the electrical (Figure 2a) and natural mechanical and thermal (Figures 2e and 2f) evoked neuronal responses in the sham control group. For instance, the top dose of LCM (100 μg) did produce a reduction of the evoked neuronal response to vF 26 *g* and 48°C heat in some of the sham control experiments, but overall no effect reached significance compared with the mean predrug baseline neuronal response.

#### Effects of systemic administration of lacosamide

Systemic administration of LCM reduced the overall neuronal response evoked by Aβ- and C-fibre activity in the MIA group; all other measures of neuronal excitability and total neuronal response attributed to Aδ-fibres were resistant to LCM’s inhibitory effects (Figure 3b). Similarly to the ‘spinal LCM’ group, significant inhibitions of the evoked neuronal responses to brush, mechanical (8 to 60 *g*) and thermal (45°C and 48°C) stimulation were seen with 3 and 30 mg/kg LCM in the MIA group (Figures 3d, 3f and 3h). In complete contrast, LCM at these doses had no significant effect on any neuronal measure in the sham control group (Figures 3a, 3c, 3e and 3g).

#### Effects of systemic lacosamide on behavioural measures of hypersensitivity

**Figure 4:**
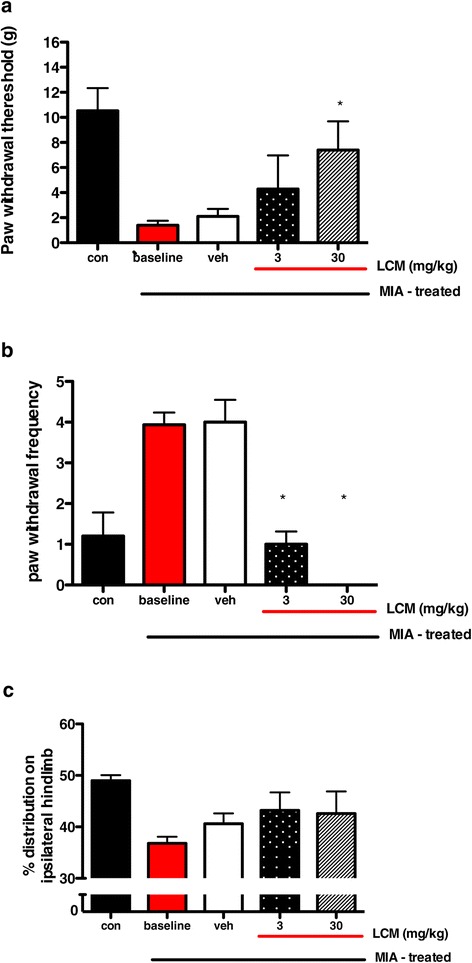
**Effects of lacosamide after monosodium iodoacetate injection.** These graphs depict the effects of lacosamide (LCM) on paw withdrawal threshold after mechanical punctate stimulation **(a)**, paw withdrawal frequency after cooling stimulation **(b)** and hind limb weight-bearing **(c)** after monosodium iodoacetate (MIA) injection. LCM at 30 mg/kg reversed the mechanical hypersensitive response. LCM at both doses reversed the hypersensitivity to cooling stimulation but had no effect on hind limb weight-bearing. * denotes significant effect (*P* < 0.05 by Kruskal-Wallis test with Dunn’s *post hoc* test) comparing LCM effect with vehicle-treated animals. Data are from five animals per group. Values are mean ± SEM.

LCM at 3 and 30 mg/kg reversed some of the behavioural measures of hypersensitivity seen in the MIA group compared with the vehicle-treated group. Treatment with LCM at 30 mg/kg significantly increased the PWT of the arthritic hind paw from 1.4 ± 0.4 *g* to 7.4 ± 2.3 *g* (Figure 4a). Treatment with the lower dose of LCM (3 mg/kg) produced some reversal of mechanical allodynia, but this did not reach significance overall (Figure 4a). LCM at both doses significantly reduced the paw withdrawal frequency to cooling stimulation of the hind paw compared with vehicle effect (Figure 4b). In contrast, ongoing joint discomfort and/or pain, as measured by hind limb weight-bearing, was not significantly improved by LCM at these doses (Figure 4c).

## Discussion

OA is a complex disease of the whole joint and typically includes destruction and degradation of the articular cartilage, subchondral bone, synovial lining and connective tissues [34]. Injection of MIA into the intraarticular space of the knee joint mimics many of the pathological signs of clinical OA, is associated with hyperexcitability [10-17,27,30] and is a well-established model for the study of osteoarthritic pain mechanisms that has also been pharmacologically validated with respect to established analgesics [35-37]. Histological assessment of the knee joint was not assessed in the present study; however, we have previously demonstrated knee joint pathology seen as cartilage loss, which is characteristic of human OA, with 2 mg of MIA [27], in accord with reports by others [26,37,38]. In the present study, MIA injection produced weight-bearing asymmetry and hypersensitivity of the ipsilateral hind paw, reflecting referred pain (secondary hyperalgesia and central sensitisation), which confirms OA pain development [39,40]. Although pain symptoms in knee OA patients are mostly associated with the area surrounding the affected joint, referred pain and tenderness also occur, implying that mechanisms of central sensitisation contribute to the pain experience [2-6]. Evidence derived from knee OA patients suggests that central sensitisation is an important contributor to chronic OA pain, and a direct link between the level of sensitisation in referred areas and clinical pain intensity experienced by OA patients has been shown [41]. Therefore, the data presented here provide an electrophysiological and behavioural correlate for the spread of sensitisation seen in OA patients and allows for the study of spinal nociceptive processing and central sensitisation mechanisms.

There is good evidence that referred pain and central sensitisation generally rely on afferent drives [5,42,43]. Thus, there is a logical basis for targeting this, and central neuronal excitability, by blocking sodium channel function to reduce action potential generation and transmission. LCM is a nonselective drug in terms of its sodium channel profile; however, it exerts ‘selective’ actions by enhancing only the slow inactivation of VGSCs, unlike other sodium channel–blocking drugs that bind to the fast inactivation state (where classical antiepileptic drugs act) [44,45]. The slow inactivation state of VGSCs can be induced by repeated neuronal firing and/or under conditions of slight or sustained membrane depolarisation, as is the case for neurones in pathological conditions such as epilepsy and neuropathy [44,46]. So, too, it is probable for OA because an increased incidence of spontaneous activity and enhanced responsiveness of joint nociceptors and dorsal horn neurones have been reported [10-17].

Our *in vivo* electrophysiological results show that the inhibitory effects of LCM are MIA state-dependent; that is, the presence of OA favours recruitment of the slow inactivated state of VGSCs because the drug had minimal effects in the sham animals at these doses. This further suggests that, if used in patients, LCM may allow physiological transmission, yet attenuate abnormal pathophysiological transmission. This is a key point because drugs such as lidocaine have to be given locally to avoid interactions with cardiac and neuronal function, but if, as the data suggest, LCM has preferential effects only on the mechanisms behind the OA pain, then oral administration is possible.

LCM reduced firing to a wide range of mechanical and thermal stimuli, when given both spinally and systemically, suggestive of a generalised state of abnormal sensitivity within the area of referred pain. It is not possible to ascertain if the drug effect occurs via reduced activity in the inflamed knee afferents and/or more directly on the abnormal spinal neurones. The latter action is supported by the ability of spinal administration of LCM to attenuate the responses, indicating that a proportion of the effect of the drug occurs via spinal actions on VGSCs located on central terminals of primary afferents and/or second-order neuronal networks.

Importantly, LCM not only acted to reduce spinal neuronal firing but also normalised the reduced mechanical thresholds induced by MIA in the behavioural studies. Systemic administration of LCM at 30 mg/kg significantly reversed the hypersensitive response to mechanical and cooling stimulation of the hind paw, in line with the inhibitory effects of this dose on the mechanical punctate and brush-evoked neuronal responses in the MIA group. The lowest dose of LCM (3 mg/kg) produced some reversal of the mechanical hypersensitive response, but this did not reach significance, which contrasts with the significant inhibition in the mechanical evoked neuronal response. This disparity between the electrophysiological data and the behavioural outcome may be explained by the fact that the level of neuronal activity remaining after LCM treatment (3 mg/kg) is still sufficient to elicit a behavioural response. It is interesting to note that LCM, via both routes of administration, was able to inhibit the electrically evoked Aβ-fibre- and dynamic brush-evoked response in the MIA group only, suggesting that LCM would likely be effective against tactile allodynia in OA, which fits well with the effects of LCM on the mechanical hypersensitive response seen in the present study. Alterations in the electrophysiological properties of Aβ-fibre low-threshold mechanoreceptors have been reported in a surgically induced model of OA [47]. This may reflect a change in sodium currents in these afferents and could underlie the preferential effect of LCM on Aβ-fibre-evoked responses in the MIA rats in the present study.

It has previously been shown that systemic administration of 30 mg/kg LCM can reverse secondary tactile allodynia [48]; however, the researchers in that earlier study only observed significant effects of LCM in MIA rats at days 3 and 7 after arthritis induction, and not on day 14, which contrasts with our findings. This discrepancy is explicable by the fact that vehicle injection produced a much greater increase in the PWT in the study by Beyreuther *et al*. [48], thus ‘masking’ any effect of LCM at that particular time point. They did, however, observe a significant antinociceptive effect of 3 and 30 mg/kg LCM on mechanical hyperalgesia at day 14 after arthritis induction, which compares well with the potent inhibition produced by systemic LCM of the suprathreshold mechanical (vF 26 and 60 *g*) evoked neuronal responses that we observed.

In contrast to the effects of the drug on mechanical hypersensitivity, LCM at both doses completely reversed the behavioural hypersensitive response to cooling stimulation, suggesting greater analgesic efficacy against cold allodynia. However, weight-bearing asymmetry was unaffected. This may indicate a lack of effect on ongoing pain or insufficient sensitivity of this commonly used measure. It is clear that LCM is able to modulate both threshold and suprathreshold responses to a range of natural stimuli, but, importantly, a lack of effect on weight-bearing does not preclude efficacy on other measures.

The observed selectivity of LCM for the MIA group may reflect an enhanced and selective responsiveness of sodium channels within nociceptive pathways in the arthritic condition, possibly due to increases in sodium channel expression [49] and/or a greater proportion of sodium channels in the conformational state favoured by LCM (that is, slow inactivation) [44]. This model of MIA-induced OA has features consistent with neuropathy, including upregulation of the neuronal damage marker cAMP-dependent transcription factor (ATF-3) in peripheral nerves that innervate the knee joint, as well as alterations in spinal cord neuroimmune cells [25-28]. Furthermore, ectopic activity within injured nerves has been demonstrated in both patients and rats [50-53]. Therefore, it is possible that ectopic activity of nociceptors in and around the OA knee joint is a source of sustained pain referral and central sensitisation seen in the MIA rats. Indeed, a greater incidence of spontaneous activity and increased firing frequency in joint-associated primary afferents has been demonstrated, as well as a greater excitability of deep dorsal horn neurones in MIA rats [10-12,14-17]. Taken together, this means that there is a far greater probability that the VGSCs present on afferent nerves in the MIA group are in the slow inactivated state, as compared with the sham control group, thus providing the functional basis for the ‘selective’ actions of LCM in targeting the nociceptive neuronal activity in the MIA group only.

LCM produced a significant and profound dose-dependent inhibition of many electrophysiological and behavioural measures of nociception after spinal or systemic administration in MIA animals. These findings align with those previously reported by Beyreuther *et al*. [48] and may predict analgesic efficacy in OA pain patients. However, these preclinical findings for LCM in models of OA pain have not positively translated to the clinic. Interestingly, the assay sensitivity of randomised controlled trials has been called into question [54]. If, for instance, the pharmacological sensitivity of OA patients with neuropathic features differs from that of other OA patients, it might explain LCM’s poor performance in clinical trials for OA in a heterogeneous population [28]. The link between disease severity and neuropathic elements to the pain suggests that the analgesic efficacy of LCM should be reassessed in clinical trials enriched with OA patients with neuropathic pain signs and symptoms. Indeed, if OA patient heterogeneity could be assessed in a manner similar to that designed by the German Research Network on Neuropathic Pain and grouped into phenotypically distinct subsets [55], then this may provide a better outcome for LCM in treating OA pain. Independently of putative neuropathy, study of OA patients with signs of central sensitisation and spreading pain could be equally informative because this could be driven by ongoing peripheral firing. In line with this hypothesis, a recent clinical trial demonstrated analgesic efficacy of oxcarbamazepine, another sodium channel blocker, in a subgroup of neuropathic patients following patient stratification according to pain phenotype [56]. The drug was effective in patients with ‘irritable nociceptors’, and this phenotype could be present in OA patients, even those without neuropathy.

## Conclusion

LCM significantly inhibited neuronal responses only in the MIA group, suggesting that changes in VGSC function play a key role in mediating the neuroplasticity seen in the OA pain state. The inhibitory effect on spinal neuronal firing aligned with the observed analgesic efficacy on behavioural measures of pain suggest that LCM may still prove worthwhile for the treatment of OA pain and merits further clinical investigation. The findings of the present study add to the large body of evidence demonstrating the importance of alterations in VGSC activity in mediating different chronic pain states and therefore advocates the continued development and evaluation of novel sodium channel–blocking drugs for the treatment of OA pain.

## Abbreviations

LCM: Lacosamide
MIA: Monosodium iodoacetate
OA: Osteoarthritis
PHN: Postherpetic neuralgia
PWT: Paw withdrawal threshold
RM-ANOVA: Repeated-measures analysis of variance
SEM: Standard error of the mean
VGSC: Voltage-gated sodium channel
